# Left Ventricular Assist Device as a Destination Therapy: Current Situation and the Importance of Patient Selection

**DOI:** 10.3390/life13041065

**Published:** 2023-04-21

**Authors:** María Melendo-Viu, David Dobarro, Sergio Raposeiras Roubin, Carmen Llamas Pernas, Candela Moliz Cordón, Miriam Vazquez Lamas, Miguel Piñón Esteban, Maria Ángela Varela Martínez, Emad Abu Assi, Rafael Pita Romero, Juan José Legarra Calderón, Andrés Íñiguez Romo

**Affiliations:** 1 Cardiology Department, University Hospital Álvaro Cunqueiro, 36213 Vigo, Spain; 2 Health Research Institute Galicia Sur, 36312 Vigo, Spain; 3 Faculty of Medicine, University Complutense of Madrid, 28040 Madrid, Spain; 4 Anaesthesiology Department, University Hospital Álvaro Cunqueiro, 36312 Vigo, Spain; 5 Nephrology Department, Regional University Hospital of Málaga, 29010 Málaga, Spain; 6 Cardiovascular Surgery, University Hospital Álvaro Cunqueiro, 36312 Vigo, Spain

**Keywords:** left ventricular assist device, destination therapy, patient selection, frailty, comorbidities, right ventricular disfunction

## Abstract

Advanced heart failure is a growing problem for which the best treatment is cardiac transplantation. However, the shortage of donors’ hearts made left ventricular assist devices as destination therapy (DT-LVAD) a highly recommended alternative: they improved mid-term prognosis as well as patients’ quality of life. Current intracorporeal pumps with a centrifugal continuous flow evolved in the last few years. Since 2003, when first LVAD was approved for long-term support, smaller device sizes with better survival and hemocompatibility profile were reached. The most important difficulty lies in the moment of the implant. Recent indications range from INTERMACS class 2 to 4, with close monitoring in intermediate cases. Moreover, a large multiparametric study is needed for considering the candidacy: basal situation, with a special interest in frailty, comorbidities, including renal and hepatic dysfunction, and medical background, considering every prior cardiac condition, must be evaluated. In addition, some clinical risk scores can be helpful to measure the possibility of right heart failure or morbi-mortality. With this review, we sought to summarize all the device improvements, with their updated clinical results, as well as to focus on all the patient selection criteria.

## 1. Introduction

Heart failure (HF) is a major problem that implies high healthcare costs in developed countries [1,2,3]. While the incidence of HF continues to grow, in a longer life expectancy population, the need for therapies for advanced stages is also increasing.

Advanced heart failure (AHF) is characterized by progressive symptoms refractory to optimal medical therapy (OMT) [4], including devices such as cardiac resynchronization therapy or percutaneous mitral valve repair. In this situation, cardiac transplantation continues to be considered the gold standard treatment.

In recent decades, heart transplantation (HT) was characterized by the refinement in donor and recipient selection. Candidates, in both situations, are often more aging, and potential recipients show worse renal function, and greater prevalence of diabetes or past malignancy when compared to historical cohorts [5]. Despite this, and according to the last report of the International Society for Heart and Lung Transplantation (ISHLT) registry, current survival is significantly longer: at 1 and 5 years is approximately 85% and 70%, respectively [5]. 

Left ventricular assist devices (LVAD) emerged in recent decades as an option for a bridge to HT, or even as destination therapy for those who are not HT candidates [6]. These devices allow short and midterm survival comparable to HT with third-generation centrifugal full magnetic levitation devices [7]; but long-term survival still favors HT. However, the increasing number of patients with AHF, with the shortage of donor hearts, makes HT unattainable for the vast majority of patients [8,9]. Thus, modern LVAD devices are preferable to just medical treatment in advanced stages when HT is not an option, as overall survival and quality of life are significantly better [10,11]. Other options, such as surgical ventricular restoration, are reserved for highly selected minorities [12]. At present, more than half of all LVAD implants are destination therapy (DT-LVAD). According to the 2022 INTERMACS registry, since 2018, the number of DT-LVAD increased from 50.4% to 66.4% in the recent era (2017–2021) and, more specifically, 81.1% in 2021 [13].

This work reviews the technological advances in recent decades and their impact on patients’ prognoses, as well as the management improvement in patient selection, in the field of LVAD as a destination therapy.

## 2. Left Ventricular Assist Devices Evolution

Current destination therapy left ventricular assist devices (DT-LVAD) are intracorporeal systems, with totally implantable pumps. Extra and para-corporeal systems were mainly relegated to biventricular support or the acute phase as a bridge to recovery or to transplant. It should also be noted that economic capacity and technological availability will condition its implementation.

**Figure 1:**
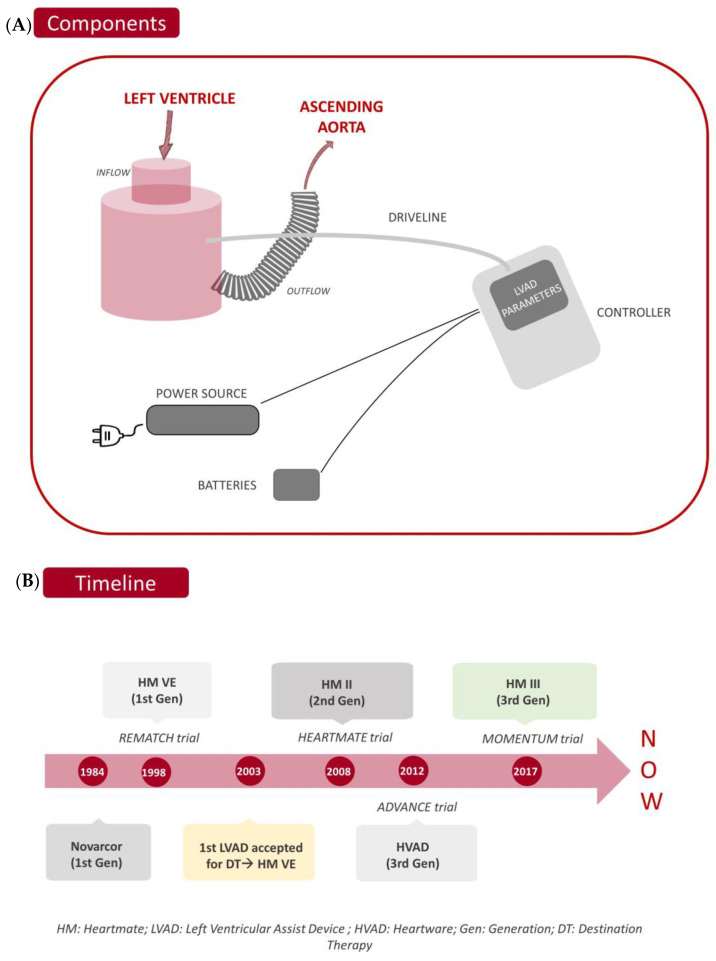
(**A**) Main components of every LVAD, (**B**) timeline of LVAD pivotal trials.

Regardless of the type of pump chosen, every LVAD shares the same components: An inflow and an outflow cannula, and a percutaneous cable (driveline) to access the control unit (usually called “controller”) and to receive power from an external supply. Finally, cable connections for connecting to external batteries, enabling patients to have greater autonomy, as well as a power source (diagram of main components seen in Figure 1A).

LVAD technology evolved from first-generation pulsatile pumps (Novacor^®^, and HeartMate VE^®^ [10]) to the continuous flow of second and third-generation LVADs (continuous improvement is shown in Figure 1B). Second-generation pumps have an axial design with a turbine system that provides a parallel flow to its rotation axis [14]. They are much smaller and quieter than first-generation LVAD. HeartMate II^®^, from Thoratec (St Jude, Abbott), has an axial pump design that was implanted in a pre-peritoneal pocket. Despite HeartMate II^®^ [15] being the best known, MicroMed-DeBakey^®^, Berlin Heart INCOR^®^, and Jarvik 2000^®^ are also included in this group. Jarvik 2000^®^, with a post-auricular connector, needs two different surgeries: one for the smallest continuous flow pump and another for the power cable skin, which is located in the left temporal bone [14,16]. It is now in the process of being evaluated as a destination therapy in adults [17], and for pediatric age in the PumpKIN trial [18]. The remaining two pumps fell into disuse. Finally, HeartMate II^®^, which showed significantly better survival [15] than HeartMate VE^®^ (58% vs. 24% at two-year follow-up), was overtaken by the later adoption of its improved version of third-generation LVAD (HeartMate III). See Table 1 for further information about every device pivotal study. 

The final group, third-generation LVAD, is the most widely used. HeartWare^®^ (HVAD), with the hybrid centrifugal flow, was not inferior to HeartMate II^®^ at the ENDURANCE trial [19], being the exchange feasible in practice [20]. However, it showed significantly higher stroke rates (regardless of what type: ischemic or hemorrhagic) than HeartMate II^®^ [21], being essential to maintaining intensive blood pressure control after implant, which was identified as an independent and strong risk factor.

Nevertheless, HeartMate III^®^ heralded a medical revolution. It is a fully magnetically levitated durable LVAD, which generates less friction in the bloodstream, and presents wider pathway gaps improving the hemocompatibility profile. In the MOMENTUM 3 trial [22], the HeartMate III^®^ was not able to show an improved overall survival compared to HeartMate II^®^ (HR 0.88, 95CI 0.61–1.16), but survival free from disabling stroke or reoperation was significantly better (see Section 3). Moreover, HeartMate III^®^ results in lower rates of gastrointestinal bleeding as well as aortic insufficiency. These results are maintained over time: with a follow-up of 5 years, when 50% of patients were still on support, event-free survival was even better with centrifugal than axial pump (54% vs. 29.7%, *p* < 0.001), especially among those DT-LVAD (54.8% vs. 39.4%, respectively, *p* = 0.005) [23]. Again, lower rates of hemocompatibility-related events (bleeding, stroke, and device thrombosis) occurred in HeartMate III^®^, even though no differences in right heart failure, arrhythmia, or infections were found. However, when biventricular support is needed, adverse events are markedly higher: bleeding and infection appearing on more than 35 and 25%, respectively [24], and, despite HeartMate III use, there was only one reported case with a survival rate of over 4.5 years [25].

There were no clinical trials that compared long-term morbidity and mortality between both centrifugal DT-LVAD devices. However, some descriptive data showed fewer complications and mortality with HeartMate III^®^ than with HeartWare^®^ [26]. In the foreseeable future, perhaps we might have another option: EVAHEART2^®^ LVAD, which is under investigation [27], designed also with large blood gaps, lower pump speeds, and an inflow cannula that does not protrude into the left ventricle. 

With hindsight, all these technological changes take the path of continuous flow, to avoid thromboembolic events, of smaller sizes maintaining bigger gaps with a simpler structure, for obtaining easier implantation with less risk of mechanical failure, and less energy consumption [28,29]. The breakthrough, expected in the next years, would enable transdermal induction to charge as a method to prevent driveline care, with its associated infectious complications [30]. Leviticus FiVAD™, a therapy pilot as a bridge to transplant in two patients [31], could be an option, if shown to be safe and durable in large-scale trials. 

## 3. LVAD as a Destination Therapy: Prognosis and Survival

Since 2003, when the Food and Drug Administration (FDA) approved the Thoratec HeartMate vented electric (VE) LVAD for long-term support in AHF patients [10], the implants continued to grow steadily, except for during the COVID-19 pandemic. From 2012 to 2021, 29,143 patients received FDA-approved durable mechanical circulatory support (MCS), and 27,314 (93.7%) of these received continuous flow durable LVAD [13,32]. Survival rates were normally better in the group of a bridge to recovery and transplant [8], presumably due to their younger age and fewer comorbidities than DT-LVAD patients. For example, the mean age of the DT-LVAD cohort at MOMENTUM 3 trial was 63 ± 12 years, and those patients usually presented with the worst renal function and higher incidence of prior revascularization surgeries [33].

The REMATCH study, a randomized trial that compared LVAD with OMT in AHF patients, played a major role in the DT-LVAD approval. Survival rates with HeartMate VE^®^ were reduced from 52% to 28% at one-year and from 29% to 13% at a two-year follow-up [10]). Some years later, the INTrEPID study was the first which compared DT-LVAD to OMT in nontransplant candidates [34]. Despite choosing inotrope-dependent patients, with ejection fraction (EF) ≤ 25% and NYHA IV for at least 3 months, higher survival rates were proven (46 vs. 22% at 6 months, and 27 vs. 11% at 12 months), with less adverse events and a substantial improvement in functional class. However, it was carried out with Novacor^®^ LVAD, again a pulsatile first-generation LVAD, and technology evolved drastically over time.

A more recent registry, the first IMACS international report, confirmed the data: survival with destination therapy LVAD was 77% at one year and 72% at 18 months of follow-up [35], while in 2018, IMACS reported a survival of 80 and 70% at one and two years, respectively [36]. Accordingly, MedaMACS registry also showed a downward trajectory of survival curves in the medical treatment group, compared to one assisted with LVAD, with longer follow-up [37]. For its part, biventricular support, reported in multicenter registries with HVAD, had worse overall survival as compared to isolated LVAD (56% and 47% at one and two years, respectively [24]). Two years of survival DT-LVAD improved from 58% with HeartMate II^®^ [19], 67.6% with HeartWare^®^ [38] to 73.2% in the case of HeartMate III^®^ [22,33]. 

Post-implant complications are quite common. According to the 2018 INTERMACS report, infection and bleeding presented with the highest incidence (40 and 35%, respectively) [36]. During the first quarter, bleeding was the most frequent complication (13.78 per 100 patient/month). Hereafter, infection, especially the one relating to driveline, and stroke appeared in 32% and 14% at 6 months. Consequently, the real problem is to define when this DT-LVAD should be implanted, which will be discussed in Section 5.

### Quality of Life and Secondary Outcomes

Clinical DT-LVAD impact in ineligible patients for transplant (INTrEPID trial) was favorable. At one year of follow-up, 85% of patients became asymptomatic or with minimal HF symptoms [34]. Similarly, an observational study, ROADMAP study, demonstrated an improvement in the functional status (measured by 6 min walk distance) with LVAD (HeartMate II^®^) compared to OMT (30 vs. 12%) [39]. No differences between HeartMate II^®^ and HeartMate III^®^ were found when comparing functional class improvement at mid-term follow-up [23].

Another interesting point in the ROADMAP study was the secondary endpoints, which showed reductions in depression screening questionnaires and improvements in patients’ quality of life (QoL)**.** This becomes particularly important in the AHF field when hospitalizations increased, and it is characterized by a debilitating course with high morbidities. Depression, as one of the most important QoL variables, was proven to be a predictor of prolonged length of hospital stay and increased short-term mortality [40]. Recently, poor health-related QoL was also identified as a strong and independent predictor of HF hospitalization and all-cause death in not-as-advanced HF stages [41].

Therefore, all efforts in AHF treatment must lead towards not only survival but QoL. In recent times, with the rising number of implants of fully magnetically levitated LVAD, and for the first time in history, MCS 5-year survival overcame 50% (51.9%) which was similar to survival in bridge to transplant group through 7 years [6]. One-year survival also improved (83%), being always highly dependent on baseline clinical situation [13], as will be explained in Section 6. LVAD also obtained a positive QoL balance [11,42]. However, clinical, psychosocial, and rehabilitation strategies were proposed to further improve QoL in LVAD patients [43,44], where caregivers play a decisive role.

## 4. LVAD Center

**Figure 2:**
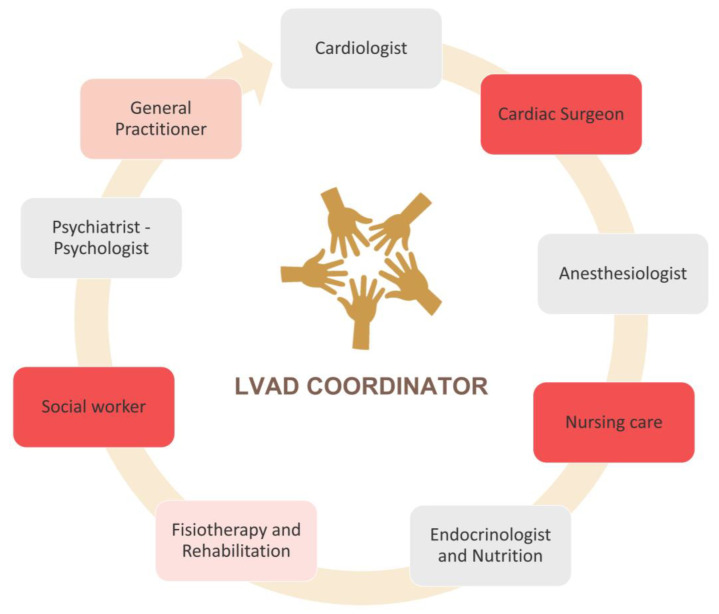
Multidisciplinary team involved in LVAD care.

Lastly, there is controversy about where DT-LVAD should be followed. The proposal for shared management between implant centers and shared-care sites could seem reasonable [45]. However, further specialized training is needed. Concerning the implant, outcomes, and results are comparable between transplant and non-transplant centers [46,47]. The health-related quality of life, the risk of death, adverse events, or rehospitalization rates remained similar after adjustment for baseline characteristics [48]. Thus, particular characteristics of the DT-LVAD program do not require a mandatory transplant program. On the other hand, a qualified team of heart failure cardiologists, cardiac surgeons, intensive postoperative care, and specialized nurses are essential (see the scheme of work in Figure 2). LVAD coordinator, probably underused at some centers, deserves special mention. Despite the heterogeneity of institutions, a coordinator is necessary for ensuring proper operation, evaluating technical information, as well as evaluating communication errors [49]. 

Moreover, the center’s portfolio must be broad with the possibility of interventional and cardiac electronic device implantation procedures if they were needed [9]. At discharge, multidisciplinary teamwork is required. This is where LVAD coordinator is key. Maintaining fluid communications with the patient’s medical practitioner as well as the primary caregiver should be the route to prevent and/or detect follow-up complications.

## 5. Patient Selection: At the Right Time

We face distinct challenges when confronted by an advanced stage of HF. The first difficulty relates to AHF definition. A European position statement tries to ascertain relevant clinical findings to identify potential risk patients to be referred, when necessary, to other more specialized HF facilities [50]. Some warnings as frequent hospitalizations or laboratory test worsening need to be carefully considered. Thus, “I NEED HELP” may be a good mnemonic rule to detect when we face AHF. EF ≤ 25% and NYHA functional class ≥ III-b may be the starting point for DT-LVAD [50].

On the other hand, to allow optimal selection of AHF patients to medical or invasive therapies, especially related to mechanical circulatory support, INTERMACS classification is mandatory [51]. Before it was created, more heterogeneity in inclusion criteria existed. REMATCH trial, for example, included patients in NYHA functional class IV during 60 of the last 90 prior days [10], whilst ROADMAP extended the scope to NYHA functional class IIIb if any recent decompensation was presented (one HF hospitalization or two unscheduled emergency visits in the last year) [39]. Conversely, while REMATCH included 65% of patients on inotropes [10], its presence, in the month before randomization, was an exclusion criterion in the ROADMAP study [39]. 

Nowadays, inotropic dependency may be considered an indication of DT-LVAD implant. However, unlike for transplants, there are no universally accepted listing criteria for LVAD, making their indication in some clinical profiles a challenge [8]. Probably the first difficult question is to wisely choose the time of implant, especially in those intermediate cases, such as the frequent flyer profile. Considering inclusion criteria for all LVAD pivotal trials, only ROADMAP and HeartWare trials included patients in a “lower” risk profile (INTERMACS 5 to 7) [19,21], where evidence could be considered positive. However, comparing the initial LVAD group with the delayed LVAD group (21.4% of patients of the OMT group) at the middle stages, no differences in survival were found. This supports the view that with the technology studied, no benefit in an early LVAD implant (without inotropes, for example) was already proven. In addition, MedaMACS registry only showed better survival in the LVAD group among patients in profiles 4 and 5, which was not the case in 6 and 7 [37] and the rest of HeartMate trials only included patients to INTERMACS 4 at most. Finally, REVIVE-IT trial failed to demonstrate a survival improvement in fewer AHF patients treated with DT-LVAD [52,53].

For all that, European expert consensus recommends implanting DT-LVAD (Class IIa, Level of evidence B) only at INTERMACS classes 2 to 4 [50]. While it is true that DT-LVAD was not demonstrated to be superior to OMT at the intermedium profile (published in 2016), and considering technological progress in the last five years, experts agree that most current trials are needed [38]. A reasonable, and more realistic, approach might be to make closer monitoring in those middle AHF cases to better identify high-risk patients that would benefit much more from such therapy [54]. Cardiopulmonary exercise tests, especially in those borderline cases, can be also helpful [6]. In addition, the hemodynamic situation must be addressed with a right heart catheterization (mean cardiac index at INTERMACS registry was 2.2 ± 0.8).

Finally, when we consider the implant of a DT-LVAD, patient choice is crucial, as seen in ROADMAP study [39]. Careful information to patients and their caregivers is required. Expert patient contact could also be used in training, especially related to daily routines which are essential in their care. It is also important to discuss life changes in three different scenarios: survival, adverse events, and last, but not least, quality of life. 

## 6. Patient Selection: Clinical Risk Factors and Checklist

**Figure 3:**
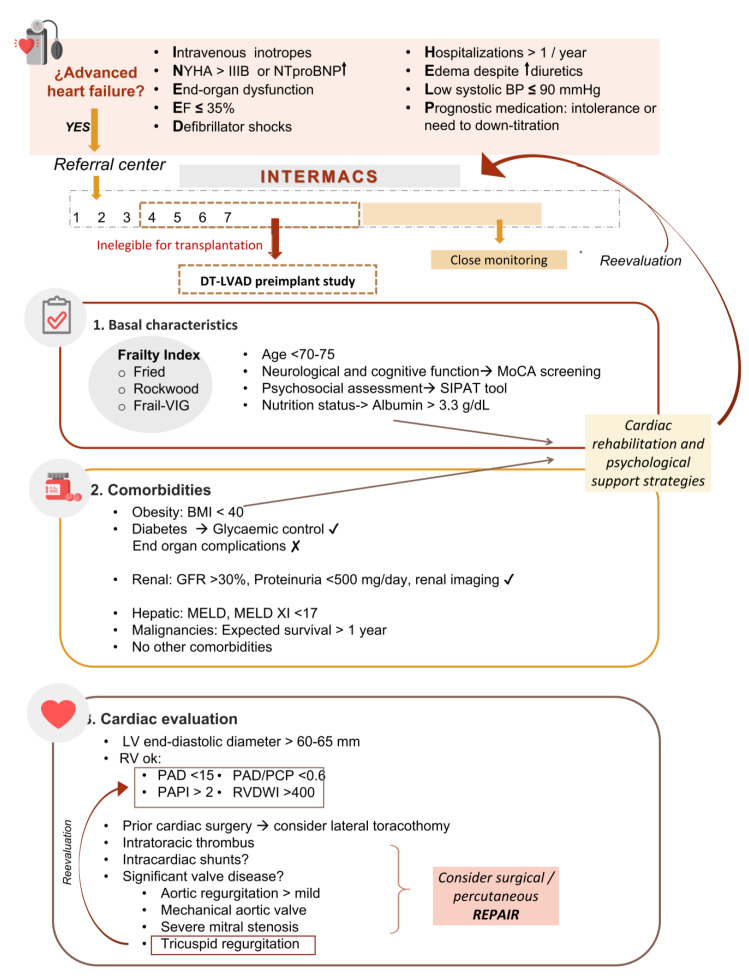
Patient selection checklist for DT-LVAD candidacy.

Careful multiorgan evaluation is needed before considering DT-LVAD candidacy. Here are the most important aspects to be considered when evaluating any patient. See Figure 3 for an essential checklist to ensure consistency in carrying out LVAD candidacy.

### 6.1. Age

In striking contrast to heart transplantation, there is no age limit for LVAD implants. Additionally, HF with reduced ejection fraction is probably far more common in elderly patients, and thanks to medical advances, they tend to arrive in a better physical and functional state.

When differences between under and over-70-year-old LVAD carriers were studied between 2013 and 2017 [55], some disparities were encountered. Understandably, as the elderly were non-transplantable, they presented greater mortality and were less likely to receive a pump exchange. They were commonly more fragile, with the worst renal function, and had undergone more cardiac surgeries before the implant, while they presented better hemodynamic profiles. Related to adverse events, stroke, and infections rates were similar, but gastrointestinal bleeding events were higher. The authors concluded that patients over 70 years old need to be more carefully evaluated before DT-LVAD implant, with special attention to fragility just as hepatic and renal function. 

On the other hand, thanks to technological refinement, a most contemporary INTERMACS analysis [56] showed similar improvements in quality of life and lower rates of complications among patients aged > 75, as contrasted with their underutilization. However, these results should be carefully considered; patients older than 75 are also under-represented (4.8% of the total) and presented with a better hemodynamic situation (better right ventricular function with less pulmonary hypertension or liver dysfunction, as well as less necessity of mechanical support). However, even then, 53% of cases required a residency facility at discharge.

According to the last registries, the average age of the LVAD group was approximately 58 ± 13 years, and only 12% were 70 or older [13,36,57]. Thus, advanced age not only is not sufficient to contraindicate LVAD use but, with new devices, is compulsory to reconsider a few carefully well-selected elderly patients as candidates for DT-LVAD [58].

### 6.2. Cardiovascular Risk Factors: Diabetes and Obesity

Cardiovascular comorbidities are quite common in this AHF population: more than one-third of patients suffer from diabetes (34% in REGALAD registry [57]), which is severe in 10–11% of cases [13]. Despite it not being a complication, its presence implicates a poor prognosis in the long-term follow-up (38.7 vs. 24.4% of mortality at 3 years) [59]. At present, only the presence of poor glycemic control or end-organ complications can be considered a relative contraindication [6].

On the other hand, obesity is also very common (28.9 ± 7.6 of body mass index in the last registries [13]), and traditionally, it was related to comorbidity, specifically infections [60]. However, recently, it was associated with higher mortality too [61]. Nonetheless, it should not be considered a contraindication nowadays [62], it is another factor to bear in mind. Finally, cardiac rehabilitation and psychosocial support strategies may be considered assuming that weight loss can be difficult, and bariatric surgery could be needed [63].

### 6.3. Renal Disfunction

Chronic kidney disease (CKD) is defined as glomerular filtration rate (eGFR) < 60 mL/min/1.73 m^2^ for ≥3 months or by the presence of albuminuria, defined as albumin to creatinine ratio > 30 mg/g in two or three spot urine specimens. CKD is a prevalent condition in patients with HF (>30% CKD, 30% microalbuminuria, and 10% macroalbuminuria), especially in AHF (54.7%, with mean creatinine level 1.3 ± 1 mg/dL in LVAD registries [13,57]), and it is related to mortality increase at 5 years follow-up [64]. Probably it is the cornerstone of the candidacy’s algorithm not only for its high prevalence but for the difficulties in its interpretation. 

When we face a pre-DT-LVAD implant study, we expect an improvement in glomerular filtration rate (eGFR) thanks to hemodynamic optimization. Two conditions coexist in cardiorenal syndrome: forward, due to low cardiac output, and backward failure, due to increased intra-abdominal pressure. However, there is considerable heterogeneity in renal behavior after LVAD implant, becoming essential to better correlate and classify a renal injury as well as predictor factors of recovery [65]. First, the presence of proteinuria usually confers a poor prognosis, being related to renal failure (32 vs. 16% in the first year) and a twofold risk of mortality [66]. However, it cannot be the only mechanism involved in diabetic nephropathy, which was also independently associated with a lack of improvement after LVAD implant [67]. Congestion laboratory profile (bilirubin, alanine aminotransferase -ALT- values), and lower eGFR were associated with better renal recovery after LVAD implant. The elderly, on the contrary, were related to worst improvement, probably due to greater kidney ischemia and fibrosis [67]. 

Some essential kidney variables should be carefully studied before deciding on DT-LVAD: 

Firstly, **glomerular function and laboratory biomarkers**. Patients with eGFR < 30 mL/min/1.73 m^2^, calculated by chronic kidney disease epidemiology collaboration (CKD-EPI) [68], or on dialysis are generally ineligible for this therapy [69,70,71] due to their high mortality (40.6% during hospitalization and 61.5% at 1-year [72]). The change in kidney function over time provides a lot of information: decline in GFR (maximum physiological expected 1 mL/min/1.73 m^2^ per year), especially if it is rapid, is strongly related to poor prognosis. High serum creatinine or urea concentrations were also proven to be independent risk factors for mortality (HR of 1.06 and 1.05 per 0.1 mg/dL and 1 mg/dL increase, respectively) at INTERMACS registry report, but neither of them is considered as a contraindication itself. However, an elevated serum urea nitrogen-creatinine ratio may be helpful to identify irreversibility [69]. Fractional excretion of sodium (FENa) determines if renal damage is due to pre-renal damage. Fractional excretion of urea-nitrogen (FEUN) is like FENa but can be used in patients with diuretics. Both are determined by dividing the quantity of sodium/urea excreted by sodium/urea in blood and multiplying by 100. Normally, a FENa < 1% and a FEUN < 38% are suggestive of pre-renal injury. So, worse outcomes were associated with a FENa above 1% [73,74] and FEUN above 35% [75,76] at the discharge of an acute HF decompensation. Finally, albuminuria or proteinuria must be addressed, as they normally reflect glomerular damage (for example, due to diabetes). Severe proteinuria (≥500 mg/day) is related to inflammation, tubulointerstitial fibrosis, and less potential recovery [77]. Secondly, **renal imaging** can be useful, along with other parameters, in defining irreversible damage. The most used, ultrasonography, permits to measure kidney and urinary tract size as well as cortico-medullary differentiation. Tomography and nuclear imaging permit the assessment of renal parenchyma, and resonance differences between interstitial inflammation and fibrosis [78]. Small kidneys, fibrotic, with poor cortico-medullary differentiation may not be recovered despite hemodynamic support, being less likely to be considered DT-LVAD candidates. Finally, in some cases, with a progressive increase in creatinine or proteinuria of unknown cause, **a renal biopsy** could be considered to rule out irreversible parenchymal damage. 

After careful evaluation, some therapeutic strategies can be useful in patients with severe renal insufficiency. In fact, aggressive treatment with inotropes and even short-term VAD prior to DT-LVAD surgery in this cohort was not related to a midterm morbi-mortality increase [79]. Even though other comorbidities must be considered, as previously described, since the coexistence with diabetes, obesity, and/or elderly make patients less suitable for candidacy. To conclude, multiparametric evaluation is needed to establish the suitability, and aggressive preoperative optimization (via improvement of cardiac output and reduction in filling pressure) in significant CKD is encouraged [6]. 

### 6.4. Liver Dysfunction 

Some similarities are shared between hepatic and renal dysfunction. Both pathological pathways, poor organ perfusion, and venous congestion, are the same in HF patients. The difference can be the better capacity of reversibility in the case of hepatic dysfunction. History of liver cirrhosis was not associated with poor prognosis in a large historic cohort of LVAD patients [80]: nor differences in infectious complications neither in hemodynamic ones (acute kidney injury, acute coronary syndrome, stroke, arrhythmias, and use of non-invasive ventilation). In addition, periportal fibrosis as an early stage of cardiac cirrhosis, shown in patients with more than five times normal transaminases or three times normal bilirubin, did not confer a worse short-term survival [81]. However, more data regarding biopsy stratification are needed, because for now, liver fibrosis is not considered a formal contraindication by itself. Bilirubin is the preferred lab test to evaluate liver function [6], but it may be insufficient to ensure a short-term prognosis. Either the model for end-stage liver disease (MELD) or MELD-XI (excluding international normalized ratio -INR- for patients in HF with oral anticoagulation) scores should chosen to stratify the risk with a cut-off point of 17 from which surgical risk can be regarded as unacceptable [82]. Finally, “irreversible” liver dysfunction constitutes a formal contraindication [6].

### 6.5. Cardiac Conditions

Prior detailed cardiac imaging is needed: significant valve diseases, intracardiac shunts, or thrombus must be discarded. A transoesophageal echocardiogram is mandatory, and tomography or resonance imaging is commonly useful. Untreated more than mild aortic regurgitation or severe mitral stenosis are absolute contraindications [6]. Biological valve replacement (with surgical correction of ascending aorta when needed) [83], closure of any septal defect, and mitral repair are the right solutions. Moreover, bioprosthetic aortic valves are preferred over mechanical ones, being advisable as a replacement when it happens. The removal of any right chamber thrombus and left atrial appendage closure, in these cases, are also needed [6]. Finally, tricuspid insufficiency should be carefully addressed, at the time of right ventricle (See Section 6.9), after fluid and hemodynamic stabilization. Tricuspid plays such an important role, that moderate or severe regurgitation makes it necessary to review the candidacy. 

Atrial or ventricular arrhythmias should be treated according to the European Society of Cardiology or the European Heart Rhythm Association, with invasive management when needed. For its part, the implantable cardioverter defibrillator (ICD) as a prevention strategy implantation (21% and 25% of cases in REGALAD and INTERMACS, respectively), and despite complications being low, was not related to benefits in mortality [84]. Thus, ICD routine implantation for primary prophylaxis before LVAD implant is not recommended [6,85].

Finally, smaller left ventricular size (cut-off point ≤ 59 mm) was related to a higher necessity of right ventricular support and worst comorbidities and survival [86]. On the other hand, prior cardiac surgery is a quite common situation (11% of cases in REGALAD registry) that, even if it is not considered a contraindication, forces us to think in a left thoracotomy approach. Some data comparing standard implantation (fully sternotomy approach) with lateral thoracotomy (with outflow graft anastomosis to the descending aorta) proved similar short-term outcomes [87]. A recent meta-analysis showed that left thoracotomy was associated with a decrease in blood transfusion or reoperation for bleeding, as well as in postoperative support requirement (inotropic or right ventricular assist devices) [88]. In the end, optimizing lung function, considering respiratory physiotherapy, and aggressive treatment of pulmonary edema are recommended before LVAD implantation [6].

### 6.6. Medical Background

Malignancies do not always implicate poor prognosis after LVAD implant. While it is true that renal cell and hematological malignancies diagnosis were related to the worst prognosis in these patients, survival is estimated at 3.5 years in most cases [89], which is more than for some AHF patients presented with medical treatment. Moreover, some experiences with surgery were published in LVAD patients with, for example, the coexistence of lung, gastrointestinal, or skin cancers [90]. Moreover, the safety of breast and hematological oncological treatments (whether they are chemo or radiotherapy) was also shown to be safe in terms of right heart failure and survival [91]. The latest European association of cardiologists and cardio-thoracic surgery consensus only recommends avoiding DT-LVAD when the malignancy expected survival is less than one year [6]. Finally, borderline indications and evaluations must be discussed in a multidisciplinary committee. 

Other comorbidities must be screened: severe peripheral vascular disease, coagulopathies, or hemostatic deficiencies, for example, are considered a relative LVAD-implant contraindication. In milder cases, in terms of avoiding perioperative hemorrhagic risk, coagulation optimization is needed. Withdrawal of vitamin K antagonists, advocating for short-acting intravenous anticoagulation as bridging is also recommended [6]. Additionally, unnecessary dual antiplatelet therapy must be removed, attending to European guidelines for myocardial revascularization. 

On the other hand, active substance abuse or active systemic (bacterial or fungal) infection, including endocarditis, are a major contraindication for any device implant, and therefore, for DT-LVAD too [6].

### 6.7. Basal Situation and Frailty

The neurological and cognitive function should be carefully studied before any DT-LVAD implantation. Some classical studies used recent (during the six prior months) transient ischemic attacks or cerebrovascular accidents as an exclusion criterion for LVAD implant [34]. However, with LVAD management development, preimplant stroke is currently present in 3.6% and 10% of patients in the INTERMACS and REGALAD registries, respectively, and other cerebrovascular conditions were found in 3.8% of patients from the INTERMACS cohort [6,92]. There is a simple and rapid screening tool (Montreal Cognitive Assessment: MoCA) that permits the identification of milder forms of cognitive impairment (score of <26 out of 30) [93]. However, in doubtful scenarios, an overall assessment is needed. 

Related to psychosocial assessment, only one predictive measure is studied in this scenario. The Stanford integrated psychosocial assessment for transplantation (SIPAT), a highly reproducible tool, was also recently associated with adverse cardiac events in the DT-LVAD follow-up. 

Frailty is a multisystemic condition that implies vulnerability in the face of stress [94,95]. As happens in other cardiovascular entities, interventional therapies are fully conditioned by the presence of frailty. This complex situation, which includes mental deterioration/dementia, nutrition status, and multiparametric assessment, is a risk factor for morbidity (longer time to extubation and hospital length of stay) and long-term mortality in LVAD patients [95]. The estimated prevalence of frailty rises to over 20% in LVAD patients [95]. Some scales were described for people older than 65, which is most of the DT-LVAD target population. The common characteristic shared by them is the multiparametric approach encompassing the cognitive and nutrition status, standing balance, strength, and cachexia (loss of muscle, measured as >5% edema-free body weight loss during the last year [4]).

In terms of nutrition, absolute body mass index (BMI) can only be helpful in extreme situations, such as values lower than 20, when it may decide a relative contraindication. However, albumin values are very helpful, associating poor prognosis with values lower than 3.3 g/dL [96] (mean albumin levels in INTERMACS and REGALAD registries are 3.4 ± 0.6 mg/dL [13] and 3.8 ± 0.6 mg/dL [57], respectively).

Currently, the Fried scale constitutes the most useful, and generalized functional approach. Values of 3 or more, out of 5, are commonly related to poor prognosis. However, there are others, such as Rockwood Clinical Frailty Scale [97], that better predict secondary outcomes (unplanned hospital admission) in elderly people, or the Frail-VIG index [98] which appeals quicker and easier.

Poor neurological and cognitive function or dementia constitute an absolute contraindication for LVAD implant. However, patients who live alone and/or suffer from depression can be carefully studied for LVAD implant, as in the case of poor mobility or frailty [6]. Physical rehabilitation, assessment of metabolic status, nutritional supplementation, and treating comorbidities are the only way to face this growing syndrome, being vitally important in borderline situations.

### 6.8. Risk Assessment

There is no universal tool to predict the postoperative risk of any DT-LVAD. Single clinical or laboratory risk markers were previously described and are some of the many pieces that comprise every risk score. However, as stated above, the expert consensus recommends a “*comprehensive risk assessment by a dedicated advanced HF team*” [6] in DT-LVAD preimplant assessment, where, naturally, multidisciplinary work is key. 

Inotrope dependent, prior cardiac surgery or stroke, lower albumin or malnutrition, lower sodium, higher blood urea nitrogen or dialysis, presence of coagulation abnormalities, higher bilirubin or MELD score, and smaller left ventricular size were related to worst prognosis [6,99,100].

At present, Seattle Heart Failure Model can be helpful to choose DT-LVAD optimal timing. It estimates up to three years of survival in medically treated advanced HF patients [101] but also allows to detect LVAD complications [102], making it advisable in lower-risk patients. Some other composite risk scores such as HeartMate2 [103], destination therapy risk score [104], or HeartMate 3 survival risk score [99], with modest results, can be also helpful in predicting short-term LVAD mortality.

### 6.9. Right Ventricular Failure

Right ventricular failure (also known as right heart failure: RHF) is a major cause of morbidity after LVAD implantation. It has been related to prolonged intensive care unit stay, poor quality of life, end-organ dysfunction, coagulopathy, and, lastly, short-term mortality [105]. 

RHF is much less prevalent among continuous than pulsatile LVAD. However, its prevalence depends on the diagnostic criteria used. From 3 to 35% of post-LVAD patients suffered from RHF at any time and severity course [106]. There is no standard definition, and the need for medical (inotropes, pulmonary vasodilators) or invasive (RVAD) treatment is usually included [105,106,107]. Nevertheless, different pharmacotherapy and device implant thresholds exist among centers and medical departments. 

The most accepted definition, as well as better related to mid-term prognosis, is the 2020 MCS-ARC definition of RHF, which unify 30 days as the cut-off point to determine early or late RVF [106,108]. Moreover, it includes clinical, laboratory, and hemodynamic factors. Some laboratory test predictors (hemoglobin, renal, or liver function) were already described in RHF [109], but as markers of end-organ function and congestion, there cannot be considered specific to RV dysfunction. High right pressures in the absence of tamponade/pneumothorax, ventricular arrhythmias, or increased left pressures are also part of the RHF “syndrome” [106], where a clinical exam is crucial [107]. Central venous pressure/pulmonary capillary wedge pressure (PCP) ratio > 0.54 were already related to poor prognosis [109,110]. At INTERMACS registry, the mean values of right atrium pressure and PCP are 12.4 ± 8.3 and 24.8 ± 9.6 mmHg, respectively, and the prevalence of RVF before LVAD is 14.2%.

There are many different RHF prognostic models based on clinical, echocardiographic and hemodynamic parameters. The Michigan model was the first published and commonly validated because of its simplicity (four binary variables: high aspartate aminotransferase -AST-, bilirubin, creatinine, and vasopressor requirement). A recent systematic review suggested that despite heterogeneity and “poor-to-modest discrimination”, probably EUROMACS score is the best one in predicting early RHF, due to its feasible applicability and external validation [110].

## 7. Conclusions

Mechanical circulatory support is a very reasonable alternative in the management of advanced HF patients who are ineligible for transplantation, improving their functional capacity. Implantable pumps became so refined that their event-free survival improved exponentially. Moreover, very specialized non-transplant LVAD centers became a reality. Currently expected survival is higher than 70% and 50% at two and five years, respectively. However, the short and mid-term prognosis is determined by careful preimplant patient selection. It is particularly important to evaluate the basal situation, with special attention to frailty and psychosocial field, renal function, hemodynamic parameters that help to predict prognosis and RVF, and finally, cardiac surgical approach. There, especially in comorbid patients and doubtful situations, a multidisciplinary shared-decision process is of special relevance. 

## Figures and Tables

**Table 1:** Pivotal trials of main devices. AHF = acute heart failure, FC = functional capacity, HM = HeartMate, HVAD = Hear Ware, OMT = optimal medical treatment, RCT = randomized controlled trial, RHF = Right heart failure, XVE = Vented electric * Reoperation to replace or repair the malfunctioning device.

RCT	Study Device	Comparator	Study Population	1st Outcome	Main Result		Survival 2 Years	Other Results
REMATCH [1], 2001	HM XVE (1st Generation)	OMT	129 AHF patients as DT-LVAD	Survival at 1, and 2 years	Survival at 1 year	52% HM XVE vs. 25% OMT	23% HM XVE vs. 8% OMT	- More QoL - More infection - More bleeding
HeartMate II [2], 2009	HM II (2nd generation)	HM XVE	200 AHF patients as DT-LVAD	Survival free from disabling stroke or reoperation *	Survival free for disabling stroke or device failure	46% HM II vs. 11% HM XVE	58% HM II vs. 24% HM XVE	- Similar QoL and FC - Lower RHF - Lower infection
ENDURANCE [3], 2017	HVAD (3rd generation)	HM II	446 AHF patients as DT-LVAD	Survival free from disabling stroke or device failure		55% HVAD vs. 57.4% HM II	60.2% HVAD vs. 67.6% HM II	- Less reoperation - More stroke (ischemic or haemorrhagic)
MOMENTUM 3 [4], 2019	HM 3 (3rd generation)	HM II	1028 AHF patients Short and long-term support	Survival free from disabling stroke or reoperation *		76.9% HM3 vs. 64.8% HM II	79% HM 3 vs. 77% HM II	- Less reoperation - Less events (major bleeding, stroke)

## Abbreviations

AHF	Advanced heart failure
ALT	Alanine aminotransferase
AST	Aspartate aminotransferase
BMI	Body mass index
CKD	Chronic kidney disease
CKD-EPI	eGFR calculated by chronic kidney disease epidemiology collaboration
DT	Destination therapy
EF	Ejection fraction
eGFR	Glomerular filtration rate
FDA	Food and drug administration
FENa	Fractional excretion of sodium
FEUN	Fractional excretion of urea-nitrogen
HF	Heart failure
HT	Heart transplantation
HVAD	HeartWare
ICD	Implantable cardioverter defibrillator
ISHLT	International Society for Heart and Lung Transplantation
LVAD	Left ventricular assist device
MCS	Mechanical circulation support
MELD	Model for End-Stage Liver disease
NYHA	New York Heart Association class
OMT	Optimal medical therapy
PCP	Pulmonary capillary wedge pressure
QoL	Quality of life
RVF	Right ventricular failure
RVAD	Right ventricular assist device
